# Construction of SHIVs expressing quaternary neutralization epitopes in Env and mimicking the neutralization phenotype of typical HIV-1 isolates

**DOI:** 10.1186/1742-4690-9-S2-P28

**Published:** 2012-09-13

**Authors:** JF Theis, A de Parseval, WJ Honnen, A Phogat, UC Ganapathi, Z Lai, L Peng, C Reichman, PL Moore, L Morris, Y Li, S Hu, A Pinter

**Affiliations:** 1 University of Medicine and Dentistry of New Jersey, Newark, NJ, USA; 2 Centre for HIV and STIs, NICD - NHLS, Johannesburg, South Africa; 3 Department of Pharmaceutics, University of Washington, Seattle, WA, USA

## Background

Infection of macaques with chimeric Simian/Human Immunodeficiency Viruses (SHIVs) provides a powerful model for HIV pathogenesis and vaccine development. A limitation of the SHIVs routinely used for vaccine studies, e.g. SHIV-1157ipd3N4, SHIV-SF162P3, and SHIV-BaL, is that they don’t express quaternary neutralization epitopes (QNEs) that are targets of broadly neutralizing antibodies like PG9, PG16, and CAP256 sera. We sought to introduce the QNEs into functional SHIVs to study how such antibodies develop during infection and help guide the creation of antigens capable of eliciting such antibodies.

## Methods

Pseudovirus neutralization assays were used to map the QNE-resistance determinants of SHIV-1157ipd3N4 Env and to test the sensitivities of mutant derivatives of SHIV-1157ipd3N4 and SHIVSF162P3 to anti-QNE antibodies. Full-length SHIVs expressing the mutant Envs were tested for growth in PBMCs, and high titre stocks from those that grew were used to infect pigtail macaques.

## Results

The double mutant SHIV1157 Env, Q170K/I192R, gained sensitivity to PG9, PG16, and CAP256 while maintaining resistance to CD4bs and anti-V3 antibodies. The full-length SHIV expressing this Env was able to replicate in macaque PBMCs while maintaining sensitivity to these antibodies, indicating there was no selection against these targets in the monkey cells. An update on the course of infection will be provided. Six changes in V2 (S164E, G166R, N167D, M169K, Q170K and N192R) were required to introduce the QNEs into SHIV-SF162P3 Env, and these dramatically increased sensitivity to anti-QNE antibodies. However, the full-length SF162P3 SHIV carrying this mutant Env failed to grow in macaque PBMCs; the basis of this growth defect is being studied.

## Conclusion

We created an infectious QNE-expressing SHIV with overall neutralization phenotypes similar to those of typical primary HIV-1 isolates. This SHIV will allow the analysis of the development of anti-QNE antibody responses during the course of infection, and should provide an improved model for HIV-1 vaccine evaluation.

